# Enhanced osteogenic proliferation and differentiation of human adipose-derived stem cells on a porous n-HA/PGS-M composite scaffold

**DOI:** 10.1038/s41598-019-44478-8

**Published:** 2019-05-28

**Authors:** Yaozong Wang, Naikun Sun, Yinlong Zhang, Bin Zhao, Zheyi Zhang, Xu Zhou, Yuanyuan Zhou, Hongyi Liu, Ying Zhang, Jianguo Liu

**Affiliations:** 1 Department of Joint Surgery, The First Hospital of Jilin University, 130021 Jilin, China; 2 Department of Orthopedics, Zhongshan Hospital, Xiamen University, 361000 Xiamen, China; 3 Department of Orthopedics, The First Affiliated Hospital of Xiamen University, 361000 Xiamen, China; 4 Medical College Xiamen University, Xiamen University, 361000 Xiamen, China; 5 Department of Medical Imaging, Zhongshan Hospital, Xiamen University, 361000 Xiamen, China; 6 Department of Oncology & Vascular Intervention Radiology, Zhongshan Hospital, Xiamen University, 361000 Xiamen, China

**Keywords:** Stem-cell biotechnology, Tissue engineering and regenerative medicine

## Abstract

This study explored the applicability, cellular efficacy, and osteogenic activities of porous nano-hydroxyapatite/Poly (glycerol sebacate)-grafted maleic anhydride (n-HA/PGS-g-M) composite scaffolds. Nuclear magnetic resonance (NMR) analyses indicated that approximately 43% of the hydroxide radicals in PGS were displaced by maleic anhydride. Resonance bands at 1036 cm^−1^ occurred in scaffolds containing nHA powders, and peak areas increased when n-HA weight increased in PGS-M-n-HA-0.4, PGS-M-n-HA-0.5, and PGS-M-n-HA-0.6 scaffolds. The n-HA/PGS-g-M composite scaffolds exhibited porous microstructure with average pore size of 150–300 µm in scanning electron microscopy (SEM) analysis. Differential scanning calorimetry (DSC) identified the glass transition temperature (Tg) as −25–30 °C, indicative of quality resilience. The modulus of compressibility increased when n-HA content increased. Interestingly, viability of human adipose-derived stem cells (hADSCs) *in vitro* and expression of the osteogenic related genes *RUNX2*, *OCN*, and *COL1A1* was enhanced in the n-HA/PGS-g-M composite scaffolds compared to those factors observed in PGS-g-M scaffolds. Finally, simulated body fluid (SBF) tests indicated more apatite deposits on the surface of n-HA/PGS-g-M scaffolds compared to PGS-g-M scaffolds. Overall, porous n-HA/PGS-g-M composite scaffolds possessed acceptable biocompatibility and mechanical properties, and they stimulated hADSC cell proliferation and differentiation. Given these qualities, the composite scaffolds have potential applications in bone tissue engineering.

## Introduction

A majority of skull defects are caused by disease or accidents, and these pose a major threat to human health. Over the years, the treatment of bone defects has mainly involved autologous graft or allograft; however, there are obvious deficiencies in these traditional methods, including a limitation in donor bone stocks for autologous grafts and immune rejection^1^. Recently, a number of biomaterials have become available for use in bone regeneration and remodelling, including three-dimensional scaffolds and membranes^2,3^. Bone regeneration through tissue bioengineering is a complex and advanced medical procedure. An ideal biomaterial for tissue bioengineering should possess certain features such as sufficient mechanical strength, desirable biocompatibility, biodegradability, good bone conduction performance, and high permeability^3,4^. Nanotechnology also plays an important role in the synthesis of scaffolds. Researchers have prepared biomaterial for tissue bioengineering using silk fibroin, collagen, biocompatible and biodegradable polymers, and hydroxyapatite (HA) using electrospinning^5^, phase separation^6^, or self-assembly methods^7^.

Poly glycerol sebacate (PGS), a strong biodegradable and biocompatible polyester applied in the context of tissue engineering, was first reported by Wang *et al*. in 2002, and this material possesses thermoset elastomeric properties^8^. Additionally, PGS exhibits a low elastic modulus (0.3 MPa) and resorption (under 4 months)^9^. To increase the mechanical strength of polymers, nano-and micro-sized particulates have been widely used in nanocomposite materials^10,11^. HA has typically been used for the induction of osteoconductivity, as a dermal filler for volume enhancement, and for the promotion of vascularization^12–14^. Bone mineral is a modified form of HA and composes up to 50% by volume and 70% by weight the teeth and bones within the human body. Therefore, it is used as a filler or coating to replace defective bone or to promote bone ingrowth in prosthetic implants^15^. The bioresorbability of nano-HA (nHA) is preferred over micro-HA due to a size of less than 100 nm. In hard tissue engineering, HA can induce bone regeneration and fixation through its bone conduction performance and desirable biocompatibility properties. The application of this material, however, is limited due to its brittle characteristics. Polymer materials remedy this limitation by increasing toughness and resilience. Synthetic polymer materials, such as poly(lactic-co-glycolic acid (PLGA) and poly- d, l-lactide-co-polyethylene glycol (PLA-PEG), possess desirable mechanical properties and are degraded directly in the body^16,17^.

PGS fumarate and nHA (PGSF-nHA) nanocomposites that are biocompatible and possess strong mechanical properties can promote cell adhesion, proliferation, and differentiation of osteoblasts in a rat calvarial bone defect model^18^. Bioresorbable and shape-memory nanocomposites will replace non-resorbable implants for non-healing soft tissue reconstruction. Natural compounds possessing a maleic anhydride (MAH) structure are a class of substances produced by certain microorganisms, and most of these compounds have been reported to possess desirable biological activity, particularly bactericidal activity^19^. Based on these reports, MAH was commonly used to improve the performance of composites such as polylactic acid, nano calcium carbonate, and others^20–22^. The main purpose underlying the use of MAH was to introduce reactive groups such as carboxyl groups, hydroxyl groups, and double bonds to alter cell adhesion, proliferation, and differentiation^23,24^. In this study, PGS grafted maleic anhydride (PGS-g-M) polymers were synthesized, and then n-HA/PGS-g-M composite scaffolds containing various weight to weight ratios (W/W) of n-HA and PGS-M (4:6, 5:5, and 6:4) were prepared and characterized by Fourier Transform Infra-Red spectroscopy (FTIR), ^1^H NMR, scanning electron microscopy (SEM), differential scanning calorimetry (DSC), thermogravimetric analysis (TGA), and a universal testing machine. Subsequently, biomedical applications of these materials in the context of bone regeneration were assessed *in vitro*.

## Results

### Chemical structure of PGS-g-M

**Figure 1:**
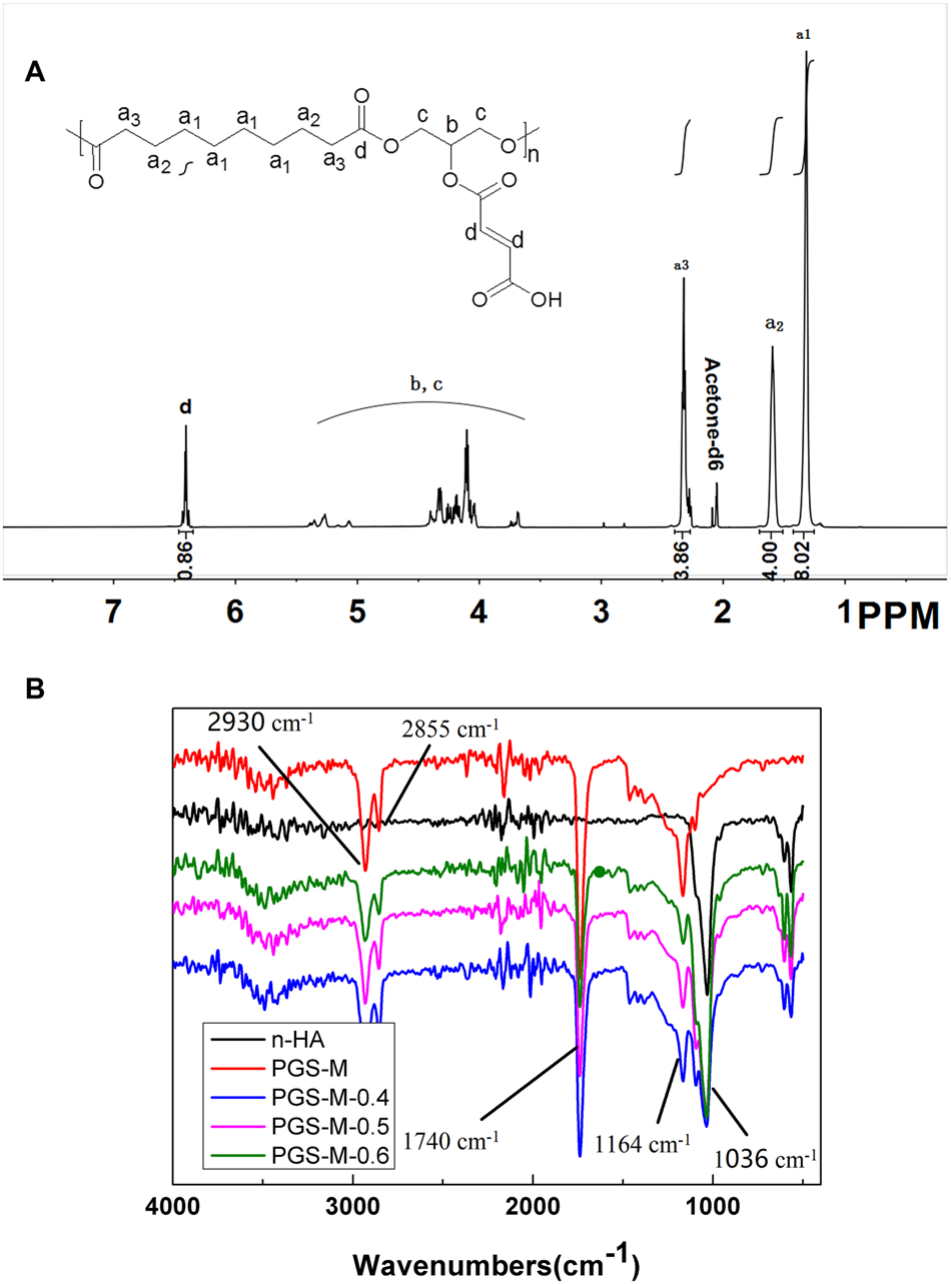
Chemical characterizations of PGS-g-M scaffolds and PGS-M-n-HA-0.4, PGS-M-n-HA-0.5, and PGS-M-n-HA-0.6 composite scaffolds. (**A**) The ^1^H-NMR spectrogram of PGS-g-M. (**B**) The FT-IR spectrogram of n-HA powder, PGS-g-M scaffolds, and PGS-M-n-HA-0.4, PGS-M-n-HA-0.5, and PGS-M-n-HA-0.6 composite scaffolds.

The chemical structure of PGS-g-M was identified by FT-IR and ^1^H-NMR spectrograms. As shown in Fig. 1A, the strong peaks of a1-a3 at 1.32 ppm, 1.59 ppm, and 2.34 ppm were attributed to the methylene protons of SA in the PGS segment of PGS-M. The weak signals of b and c at 3.69–5.39 ppm indicated the protons of glycerol units in PGS. The sharp peak of d at 6.86 ppm was attributed to the protons of C=C in the MAH units of PGS-g-M. The percent grafting was approximately 43% according to the results of ^1^H-NMR, suggesting that 43% of the hydroxide radicals in PGS were displaced by MAH. FT-IR spectroscopy displayed peaks at 2930 cm^−1^ and 2855 cm^−1^ that were attributed to alkane groups of PGS (Fig. 1B). The strong peak at 1740 cm^−1^ indicated the stretching band of C=O, and the peak at 1164 cm^−1^ was attributed to the stretching band of C-O, indicating ester linkages.

### Characterizations of scaffolds

As shown in Fig. 1B, the resonance bands at 1036 cm^−1^, which indicated the C-O of alcohol group, could not be observed in the blank PGS-g-M scaffolds but were observed in other scaffolds with nHA powders. The peak area increased when the weight of n-HA increased in PGS-M-n-HA-0.4, PGS-M-n-HA-0.5, and PGS-M-n-HA-0.6 scaffolds.

**Figure 2:**
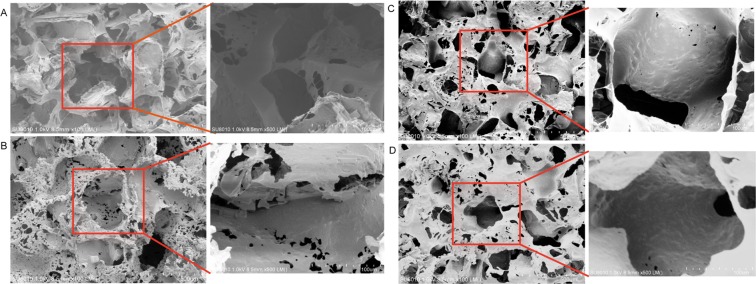
SEM images of the PGS-g-M scaffolds (**A**) and the PGS-M-n-HA-0.4 (**B**), PGS-M-n-HA-0.5 (**C**), and PGS-M-n-HA-0.6 composite scaffolds (**D**).

The SEM images indicated that the n-HA/PGS-g-M composite scaffolds exhibited a porous microstructure with an average pore size of 150–300 µm (Fig. 2). When the SEM magnified the composite scaffolds two hundred times or five hundred times, an interconnected pore structure was observed. At a magnification of 1500×, many small pore structures in the sidewall of the different scaffolds due to solvent evaporation were observed, which provided further evidence that the scaffolds possess an interconnected pore structure. Additionally, the average pore size is negatively related to the W/W of n-HA/PGS-g-M. The porosities of the blank PGS-g-M, PGS-M-n-HA-0.4, PGS-M-n-HA-0.5, and PGS-M-n-HA-0.6 scaffolds were 90.34 ± 1.02%, 90.13 ± 0.24%, 88.21 ± 0.77%, and 84.56 ± 1.54%, respectively. These quantitative results were similar to the results observed in the SEM images. These results indicated that the W/W of n-HA/PGS-g-M significantly altered the porosities of scaffolds.

**Figure 3:**
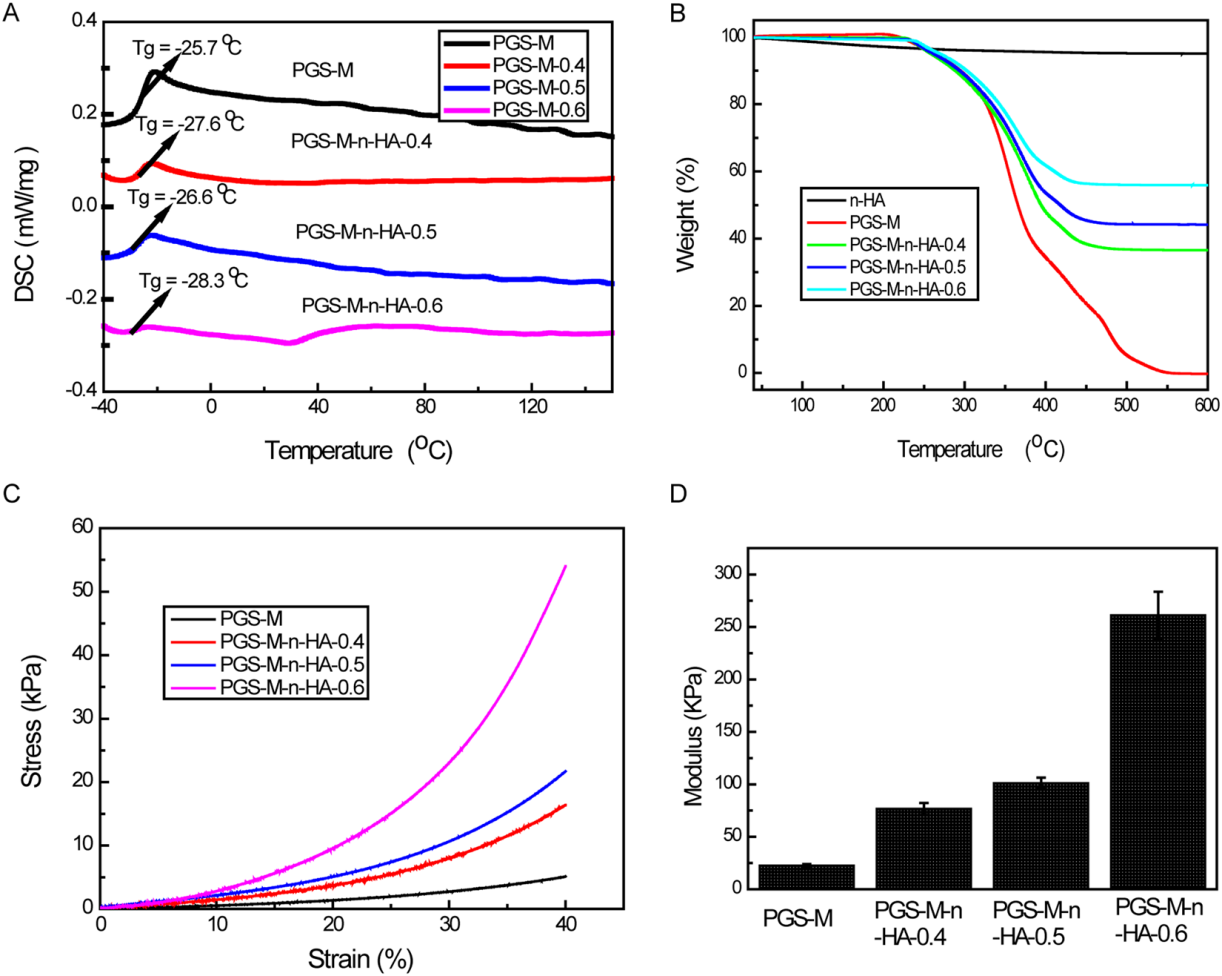
Physical characterizations of PGS-g-M scaffolds and PGS-M-n-HA-0.4, PGS-M-n-HA-0.5, and PGS-M-n-HA-0.6 composite scaffolds. (**A**) Differential scanning calorimetry (DSC) curves; (**B**) thermogravimetric analysis (TGA) curves; (**C**) the stress-strain curve; (**D**) the modulus of compressibility graphs.

The thermal properties of the scaffolds are indicated in Fig. 3. The Tg values of these scaffolds were between −25 °C and −30 °C, indicating good resilience. Additionally, crystalline peaks and melting peaks were not observed from −50 °C to 150 °C, suggesting that these scaffolds were not crystalline at 25 °C or at 37 °C (Fig. 3A). For the blank PGS-g-M scaffold, the quality change was close to 100% at 600 °C, while the quality change was only less than 5% for n-HA powder at 600 °C. The quality change was higher when the W/W of n-HA/PGS-g-M was lower (Fig. 3B). The TGA curve indicated that the W/W of n-HA/PGS-g-M was consistent.

Compressive measurements were used to evaluate the mechanical properties of n-HA/PGS-g-M composite scaffolds. In Fig. 3C, the compressive strengths of scaffolds increased when the content of n-HA increased. Similarly, the modulus of compressibility increased when the content of n-HA increased (Fig. 3D). Together, these results indicated that n-HA/PGS-g-M composite scaffolds possessing interconnected pore structure were successfully prepared.

### *In vitro* cell proliferation of scaffolds with different W/W rate of n-HA/PGS-g-M

**Figure 4:**
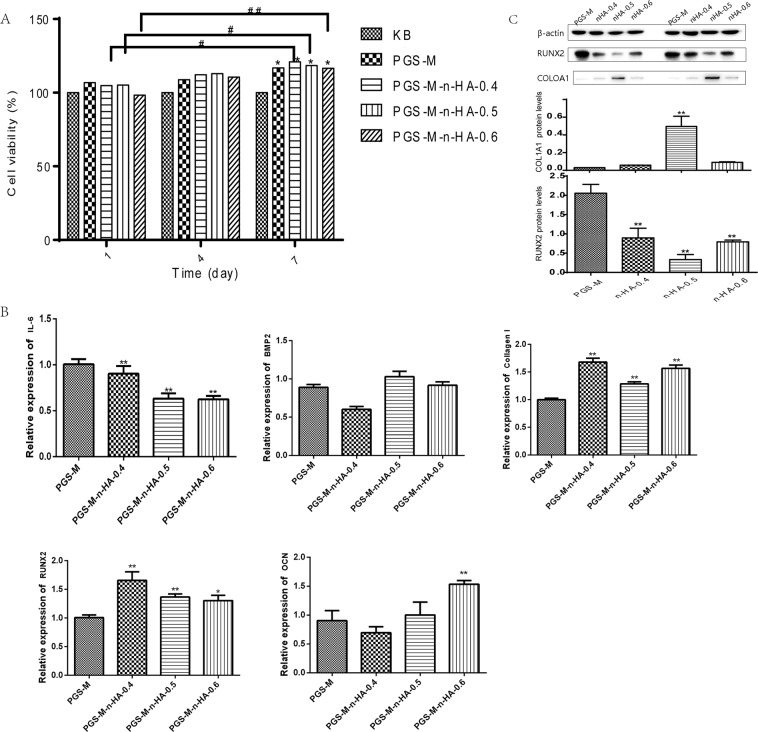
The biomedical applications of PGS-M-n-HA-0.4, PGS-M-n-HA-0.5, and PGS-M-n-HA-0.6 composite scaffolds in bone regeneration *in vitro*. (**A**) the effects of these scaffolds on cell proliferation in human adipose-derived stem cells (hADSCs); (**B**) the expression levels of proinflammatory factor IL-6, and the expression levels of genes associated with osteoblast differentiation by qRT-PCR; The levels of RUNX2, OCN proteins by western blot (**C**) and immunohistochemical staining (**D**).

To investigate the effects of these scaffold properties on cell proliferation, hADSC cells were seeded onto the scaffolds. The cell viability significantly increased at 7 days compared with that at 1 day when scaffolds with various W/W ratios of n-HA/PGS-g-M were used, with the exception of PGS-M scaffolds (all P < 0.05, Fig. 4A). At 7 days, cell viability significantly increased in the blank PGS-g-M, PGS-M-n-HA-0.4, PGS-M-n-HA-0.5, and PGS-M-n-HA-0.6 scaffolds when compared to cell viability of the blank cells (all P < 0.05).

### Inflammation response and osteoblast differentiation of hADSCs on the composite scaffolds

The inflammation response played a key role in tissue bioengineering. In this study, the expression levels of proinflammatory factor IL-6 were reduced in PGS-M-n-HA-0.4, PGS-M-n-HA-0.5, and PGS-M-n-HA-0.6 composite scaffolds compared to those observed in PGS-g-M scaffolds (P < 0.01, Fig. 4B). When compared to PGS-g-M scaffolds, PGS-M-n-HA-0.4 and PGS-M-n-HA-0.5 composite scaffolds significantly induced the expression levels of genes associated with osteoblast differentiation, including *RUNX2* (P < 0.05) and *COL1A1* (P < 0.01). PGS-M-n-HA-0.6 composite scaffolds induced increased expression levels of RUNX2, OCN (both P < 0.05), and COL1A1 (P < 0.01) (Fig. 4B). The levels of RUNX2 and OCN proteins were also increased in these composite scaffolds (Fig. 4C and Supplementary Fig. 1) and the immunohistochemical results were consistent with the other observations (Supplementary Fig. 2).

### Calcium phosphorus (Ca–P) precipitation on the surface of scaffolds

**Figure 5:**
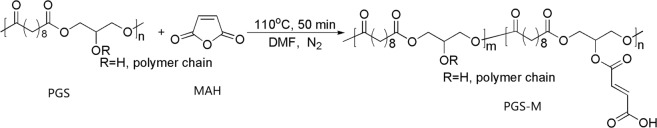
SEM images of scaffolds in simulated body fluid (SBF) from 1 week to 4 weeks. The upper left corner: magnification of SEM images.

Figure 5 shows the surface SEM morphology of the apatite layer on each scaffold when immersed in SBF for 4 weeks. Numerous smaller apatite deposits were observed on the surface of n-HA/PGS-g-M scaffolds when compared to PGS-g-M scaffolds; however, the number of apatite deposits on the scaffolds was not significantly different when nHA content increased in the composite scaffolds. Additionally, the results of EDS analysis indicate that a Ca-P chemical element was detected on the surface and porous layer of the four scaffolds, indicating that PO_4_^3−^ and Ca^2+^ ions can pass through the porous layers and form apatite structure on the surfaces (Supplementary Fig. 3).

## Discussion

PGS has been used in a large number of biomedical applications including tissue engineering and drug release due to its strength and to its biodegradable and thermoset elastomeric properties^25^. The purpose of our present study was to explore the structural characterization, cellular efficacy, and osteogenic activities of porous n-HA/PGS-g-M composite scaffolds. Wang *et al*. reported that the intense C=O stretch in ester bonds of PGS in FT-IR spectroscopy was located at 1740 cm^−1^, while the C-O stretching bond was present at 1164 cm^−1^. These findings indicated that PGS is a polyester^8^. Similarly, our results revealed peaks of alkane groups at 2930 cm^−1^ and 2855 cm^−1^, the stretching band of C=O at 1740 cm^−1^, and the stretching band of C-O at 1164 cm^−1^, which indicated ester linkages in the PGS polymer. Additionally, ^1^H-NMR spectra of PGS displayed methylene peaks at 1.30, 1.62, and 2.35 ppm that were attributed to the methylene protons of SA in PGS, while peaks observed at 4.05–5.30 ppm indicated the protons of the glycerol units in PGS^26^. These findings are similar to our results. Additionally, the sharp peak of d at 6.86 ppm was attributed to the protons of C=C in the MAH units of PGS-g-M. Taken together, our observations of FT-IR and ^1^H-NMR spectrum indicated that PGS-g-M scaffolds were successfully prepared.

Our examination of the porosity of the scaffolds revealed that the porosity was greatly decreased by the introduction of nHA into the polymer. These results are consistent with those of SEM images. This phenomenon was also consistent with a study by Huang *et al*.^27^. All of the scaffolds possessed a high porosity of >84%, and this aided in cell adhesion and proliferation^27,28^. In the present study, the compressive strengths of scaffolds increased when the content of n-HA increased in the n-HA/PGS-g-M composite scaffolds. As mentioned above, PGS is an elastomeric polymer, and HA exhibits bone conduction performance and desirable biocompatibility properties. Thus, the addition of HA reduced the elastomer of PGS and increased the strength. The results were consistent with previous studies that that demonstrated that increasing HA improved the compressive strength^29,30^. The mechanical properties were consistent with the structural observations that the elasticity for PGS-M-n-HA-0.6 and −0.4 were lower than that of the PGS-M. This phenomenon may result from the presence of the stiff hydroxyl caused by the higher incorporation of nHA groups that prevent C-O bending. The copolymers with high mechanical strength enhanced the applicability for hard tissue engineering. Additionally, the compressive modulus of n-HA/PGS-g-M composite scaffolds is enhanced when HA contents are increased. In a previous study, the compressive modulus of PGS was demonstrated to be similar (0.024 MPa) to that of PGS-g-M^31^. Hence, n-HA/PGS-g-M composite scaffolds may prove useful for hard tissue engineering.

n-HA/PGS-g-M composite scaffolds also enhanced cell viability and promoted the expression of osteogenic related genes *in vitro*. The expression of osteogenic related genes detected by qRT-PCR in bone tissue engineering has been reported by some researchers. *RUNX2* has been demonstrated to strongly influence the differentiation process of human mesenchymal stem cells into osteogenesis in the early stage of development^32^. The relative expression of the osteogenic marker *RUNX2* significantly increased during osteogenic differentiation when human mesenchymal stem cells were seeded into organic/inorganic hybrid scaffolds that contained functional rhBMP-2^33^. Similarly, PGS-M-n-HA-0.4 and PGS-M-n-HA-0.5 composite scaffolds significantly induced the expression levels of genes associated with osteoblast differentiation such as *RUNX2* and *COL1A1*. mPCL-CaP composite scaffolds up-regulated *OCN* and *OPN* expression^34^. On the biphasic Ca-P scaffolds coated with HA-PCL composites, overexpression of *RUNX2* and *COL1A1* indicated that these scaffolds induced the differentiation of primary human bone-derived cells^35^. Therefore, PGS-M-n-HA-0.4 and PGS-M-n-HA-0.5 composite scaffolds may prove useful for bone tissue engineering.

To investigate the response process and biological activity of scaffolds, SBF was used in this study, as it is a quick and easy assay possesses ion concentrations and a pH that are similar to those encountered under physiological conditions^36^. SBF has been used to evaluate the potential mineralization abilities of implant material for bone tissue engineering by previous researchers^37,38^. The formation of apatite on synthetic materials is induced, as functional groups present a negative charge and form apatite nuclei through the formation of amorphous Ca-P^39^. It has been reported that a greater number of apatite nuclei form on chitosan/nHA composite scaffolds than that of chitosan-only scaffolds due to the ability of nHA particles to act as nucleation sites^37^. The Ca and P ions present in the SBF can be spontaneously assembled into the surrounding apatite core once the apatite core is formed. Additionally, Blaker *et al*. reported that there was significant HA layer formation on the surface of PDLLA/Bioglass bone scaffolds after immersion in SBF solution for 70 days^40^. Similarly, apatite layers were found on the surface SEM morphology of the porous n-HA/PGS-g-M composite scaffolds when immersed in SBF for 4 weeks, suggesting that the formation of apatite on n-HA/PGS-g-M composite scaffolds could provide a marker for bioactivity. Additionally, more apatite deposits were observed on the surface of n-HA/PGS-g-M scaffolds when compared to those observed on PGS-g-M scaffolds. It should be noted that the number of apatite deposits was not significantly different when nHA content was increased in the composite scaffolds. Therefore, n-HA/PGS-g-M scaffolds were more effective in imparting bioactivity to the material than was the control scaffold.

In the present study, porous n-HA/PGS-g-M composite scaffolds with desirable biocompatibility, strong mechanical properties, and bioactivity have been successfully fabricated. Compared to PGS-g-M scaffolds, n-HA/PGS-g-M composite scaffolds induced elevated cell proliferation and expression levels of osteogenic related genes while causing little inflammatory response. Therefore, these n-HA/PGS-g-M composite scaffolds may be potential candidates for application in bone regeneration and remodelling.

## Materials and Methods

### Chemicals

Sebacic acid (SA) and anhydrous N,N-Dimethylformamide (DMF) were both purchased from J&K Scientific Ltd. (Beijing, China). Glycerinum was purchased from Sigma-Aldrich Chemical Co. (St. Louis, Missouri, USA), and nHA (H97%, <100 nm) was purchased from Aladdin (Shanghai, China). Acetone for HPLC was purchased from Yonghua Chemical Co. (Changshu, Jiangsu, China). Maleic anhydride (MAH) was purchased from TCI (Shanghai, China).

### Synthesis of PGS

The synthesis of PGS was modified based on a previous procedure^8^. Briefly, 20.2574 g of SA and 9.2037 g of glycerol were placed in a three-necked flask and melted at 135 °C. Then, the mixture was stirred with nitrogen for 24 h, and this was followed by vacuuming for 48 h. After the reaction system was cooled to room temperature, PGS was obtained.

### Preparation and characterization of PGS-g-M

**Figure 6:**
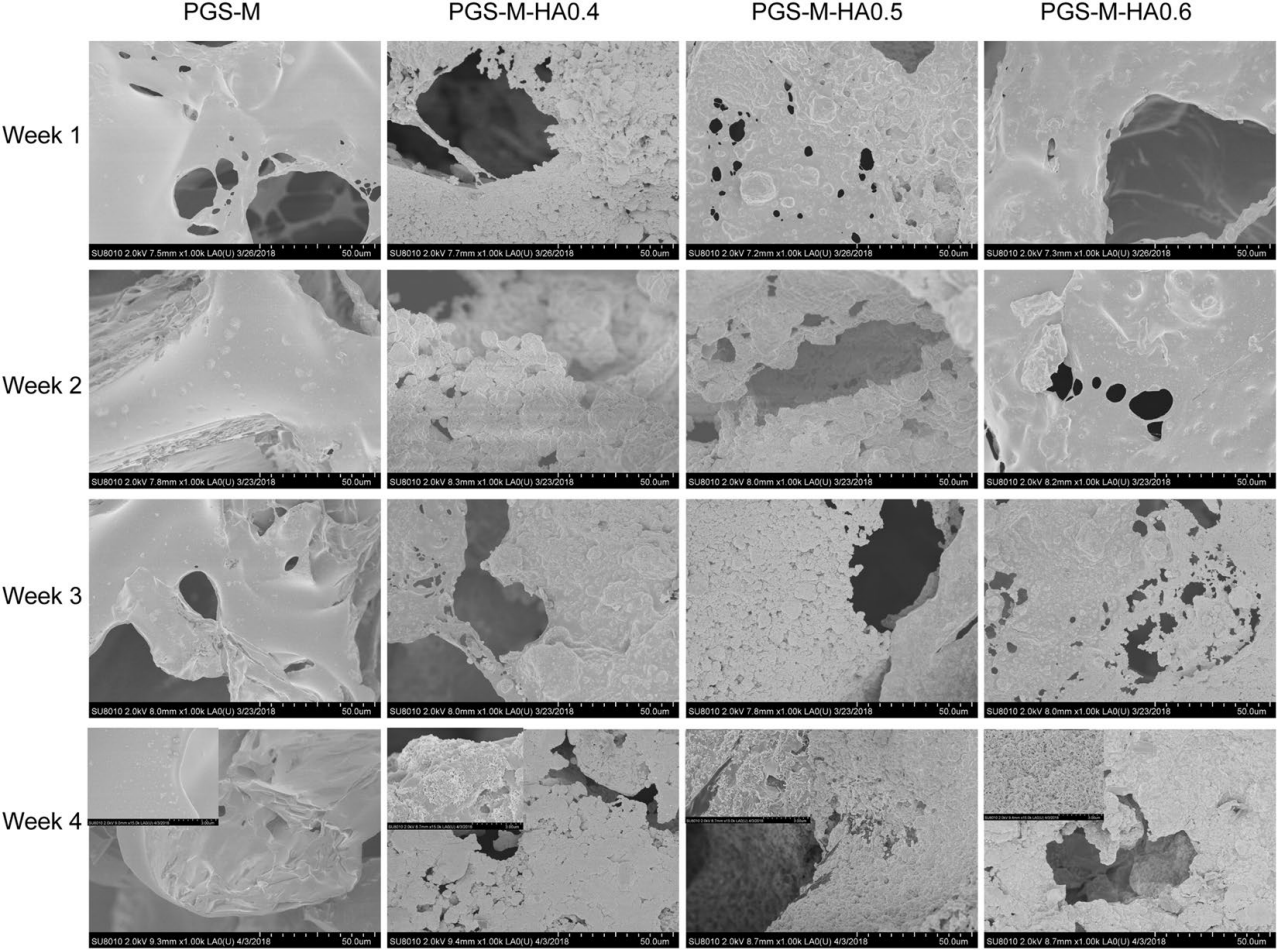
The reaction scheme for the synthesis of PGS-g-M. PGS-g-M, poly (glycerol sebacate)-graft-Maleic anhydride.

The reaction scheme for the synthesis of PGS-g-M is shown in Fig. 6. Mixtures of PGS (2.6668 g), MAH (2.0263 g), and anhydrous DMF (2.7 mL) were reacted at 110 °C under N_2_ for 50 min before 150 mL of deionized water was used to obtain the precipitate. Then, the precipitate was dissolved into acetone and washed again with 150 mL of deionized water. The above procedure was repeated twice. The faint yellow sticky solid appeared under a vacuum air-removed system (4 mbar) after 24 h. PGS-M was identified by ^1^H NMR (Bruke, 600 MHz, Germany) in deuterated acetone solution and by Fourier Transform Infra-Red spectroscopy (FTIR, Nicolet 6700, Madison, WI). The range of FTIR analysis was 400–4000 cm^−1^. The resolution of the instrument and the number of scans were 4 cm and 32, respectively.

### Preparation of n-HA/PGS-g-M composite scaffold

Firstly, a cylindrical mould (inner diameter of 4 cm) was prepared by a strong magnet. Then, sodium chloride particles with a diameter of 150–300 µm were spread over the entire mould. The mould was transferred to a relative humidity environment for 1.5 h. Third, the sample was vacuum dried at 120 °C, and the mould was removed to prepare a salt mould. Fourth, PGS-M and acetone were mixed to prepare a 280 g/L solution, and this solution was divided into 4 equal portions. Fifth, an appropriate amount of n-HA was added to the each acetone solution, bringing the final n-HA/PGS-M weight to weight ratio (W/W) to 0 (control: PGS-M), 4:6 (PGS-M-n-HA-0.4), 5:5 (PGS-M-n-HA-0.5), or 6:4 (PGS-Mn-HA-0.6). Sixth, after ultrasonic treatment for 20 min, the salt mould was soaked into the mixed solution. After evaporating, it was placed in a vacuum oven and crosslinked at 150 °C under a pressure of 1 Torr for 24 hours. Finally, the mould was washed with distilled water, and a porous tissue engineering scaffold was obtained by freeze drying for 48 h. The chemical characteristics of the scaffolds were determined by FTIR.

### Surface morphological characteristics and porosity of the scaffolds

The surface morphology and microstructure of n-HA/PGS-M composite scaffolds were both observed by a SEM (Hitachi, SU8010, Tokyo, Japan). Fluid-discharge therapy^41^ and the liquid displacement method were both used to calculate the porosity of the scaffolds, was used. Briefly, a known amount of ethanol (volume V1) was decanted into a graduated cylinder. Then, the n-HA/PGS-M composite scaffold was also placed in the same graduated cylinder, and the ethanol volume (V2) was recorded after the scaffold was completely infiltrated. Subsequently, the scaffold sample saturated with ethanol was removed, and the volume of ethanol in the graduated cylinder was defined as V3. The porosity of the scaffolds was calculated according to the following formula:

$${\rm{Porosity}}\,( \% )=({\rm{V1}}-{\rm{V3}})/({\rm{V2}}-{\rm{V3}})\times 100.$$

### Differential scanning calorimetry (DSC) and thermogravimetric analysis (TGA)

The thermal properties of the scaffolds were characterized by a DSC (204F1, Netzsch, Germany). The temperature program heated at a ratio of 10 °C/min from room temperature to 150 °C and cooled to −50 °C, and then again heated back to 150 °C. Thus, the glass transition temperature (Tg) was automatically detected. Additionally, thermo-gravimetric analyses were performed using a Q5000IR (TA, USA) under air atmosphere at a rate of 10 °C/min from 40 °C to 600 °C.

### Mechanical test

A universal testing machine (E42, MTS, USA) possessing a 25N sensor was used to test the mechanical properties of PGS-M and PGS-M-n-HA scaffolds. Each sample was compressed to 40% at a rate of 1.00 mm/min using a preload force of 0.01N. The compressive modulus was calculated from the instrument software.

### Cell Counting Kit-8 (CCK-8) assay

Human adipose-derived stem cells (hADSCs) were cultured in Minimum Essential Medium (MEM). The blank PGS-g-M, PGS-M-n-HA-0.4, PGS-M-n-HA-0.5, and PGS-M-n-HA-0.6 scaffolds (all 1 mm × 5 mm) were sterilized and co-cultured with MEM overnight. After the MEM was removed, these scaffolds were seeded with hADSCs (2 × 10^4^/ well) by pipetting of the cell suspension onto the scaffolds, and they were cultured in 24-well plates with 1 mL of MEM under conditions of small amplitude vibration. The MEM was changed every 3 days, and hADSCs treated without any scaffolds were defined as the control group. After 1, 4, and 7 days, 100 µL of 5-mg/mL CCK-8 solutions were added into each well and incubated for 2 h in the dark. The optical density (OD) value of each well was measured at 450 nm using a microplate reader. The cell viability was evaluated using the following equation:

$${\rm{Cell}}\,{\rm{viability}}\,( \% )=({{\rm{OD}}}_{{\rm{test}}}-{{\rm{OD}}}_{{\rm{blank}}})/({{\rm{OD}}}_{{\rm{control}}}-{{\rm{OD}}}_{{\rm{blank}}})\times 100 \% .$$

### Quantitative real-time quantitative PCR (qRT-PCR) assay

The hADSCs (1 × 10^5^ cells) were seeded onto these scaffolds in 6-well plates for 14 days. Then, qRT-PCR was performed to estimate the expression levels of genes associated with osteoblast differentiation (e.g. *RUNX2*, *OCN*, *COL1A1*, and *BMP2*) and inflammation-associated genes (e.g. *IL-6*). Briefly, the total RNA was extracted. Then, the single-stranded cDNA was synthesized using 4 µL of 5× PrimeScript RT Master Mix (TaKaRa Biotechnology, Dalian, China), 0.5 µg of total RNA, and RNase-free water based on the product instructions. Subsequently, RT-PCR was performed using SYBR Premix EX Taq and an ABI 7500 Real Time PCR System (7900HT FAST, Applied Biosystems) under 50 °C for 3 min, 95 °C for 3 min, and 40 cycles of 95 °C for 10 s and 60 °C for 30 s. The primers specific to each gene are shown in Table 1. Amplifications were performed in triplicate, and the relative expression levels of genes were calculated according to 2^−ΔΔCT^ method^42^.

**Table 1:** The primers in real-time quantitative PCR assay.

Primers	Sequence (5′-3′)
human-GAPDHF	AGAAGGCTGGGGCTCATTTG
human-GAPDHR	AGGGGCCATCCACAGTCTTC
IL-6-hF	ATGAGGAGACTTGCCTGGTG
IL-6-hR	GCATTTGTGGTTGGGTCAGG
RUNX2-hF	CCGCCTCAGTGATTTAGGGC
RUNX2-hR	GGGTCTGTAATCTGACTCTGTCC
BMP2-hF	GAACGGACATTCGGTCCTTG
BMP2-hR	GCAACGCTAGAAGACAGCG
COL1A1-hF	CTGGCCTCCCTGGAATGAAG
COL1A1-hR	GGCAGCACCAGTAGCACC
OCN-hF	GGATGACCCCCAAATAGCCC
OCN-hR	CTTGGACACAAAGGCTGCAC

hF, human forward primer; hR, human reversed primer.

### Western blot

After the hADSCs (3 × 10^5^ cells) were seeded onto scaffolds in 6-well plates for 14 days. Briefly, total protein was extracted and the protein samples were mixed in a 5× loading buffer and loaded into SDS-PAGE following by transferring to polyvinylidene difluoride (PVDF) membranes, blocking with 5% skim milk at 37 °C for 1–2 h, and washing six times (5 min per time) with 1× PBS-T buffer (1000 mL of 1× PBS and 1 mL of Tween-20). Subsequently, the membranes were incubated with the corresponding primary antibodies at 4 °C overnight, and this was followed by incubation with secondary antibody labelled with horseradish peroxidase at room temperature for 2 h. Chemiluminescence was captured by a Millipore ECL system.

### Immunofluorescence

The hADSCs (3 × 10^5^ cells) were seeded onto scaffolds in 6-well plates for 14 days, and the expression of RUNX2 and OCN in hADSCs cells was visualized by immunofluorescent staining. The cell samples were fixed with 4% paraformaldehyde and immersed into PBS three times (3 min/time), and then they were transferred into a 0.5% Triton X-100 PBS solution at room temperature for 20 min. After blocking with goat serum, the primary antibody against RUNX2 (1:50, mouse monoclonal antibody) and OCN (1:50, mouse monoclonal antibody) was added at 4 °C overnight. Subsequently, goat anti-mouse IgG (H + L) cross-adsorbed secondary antibody (1:200, Alexa Fluor 594) was incubated at 20–37 °C for 1 h in the dark. Finally, the 4,6-diamidino-2-phenylindole (DAPI) was added, and this was incubated in the dark for 5 min. The images were captured by an inverted fluorescence microscope (IX73, Olympus, Japan).

### Bioactivity of scaffolds in simulated body fluid (SBF)

The SBF was prepared based on the following steps^43^. First, 800 mL deionized water was taken in a 1000-mL beaker. Then, 11.994 g NaCl, 0.525 g NaHCO_3_, 0.336 g KCl, 0.342 g K_2_HPO_4_·3H_2_O, 0.4575 g MgCl_2_·6H_2_O, 0.417 g CaCl_2_, 0.1065 g Na_2_SO_4_, and 9.086 g NH_4_C(CH_2_OH)_3_ were added to the beaker successively. The temperature was maintained at 36.5 °C. The pH was adjusted to between 7.2–7.4. Finally, the volume was adjusted to 1000 mL. The scaffolds were sequentially immersed in SBF at 37 °C for 4 weeks. The SBF was changed every three days, and the surface morphologies of composite scaffolds were observed using SEM. Additionally, chemical analysis on the surface of each scaffold was performed by energy-dispersive X-ray spectroscopy (EDS) coupled with SEM.

### Statistical analysis

Data were analysed by SPSS 19.0 software (SPSS Inc., USA) and presented as mean ± standard deviation. The differences among multiple groups were determined using one-way ANOVA followed by the Fishers least significant difference (LSD) method. Statistical significance was considered at P < 0.05.

## Supplementary information

Supplementary figures

## Data Availability

The datasets used and analysed during the current study are available from the corresponding author upon reasonable request.
