# Supplementary material for: Enhanced osteogenic proliferation and differentiation of human adipose-derived stem cells on a porous n-HA/PGS-M composite scaffold
Source: Sci Rep. 2019 May 28;9:7960. doi: 10.1038/s41598-019-44478-8 (PMC6538636; doi:10.1038/s41598-019-44478-8)
Supplement: Supplementary file 1 — Supplementary figures [file 41598_2019_44478_MOESM1_ESM.pdf]

Enhanced osteogenic proliferation and differentiation of human adipose-derived stem cells on a porous n-HA/PGS-M composite scaffold

Yaozong Wang, Naikun Sun, Yinlong Zhang, Bin Zhao, Zheyi Zhang, Xu Zhou, Yuanyuan Zhou, Hongyi Liu, Ying Zhang, Jianguo Liu

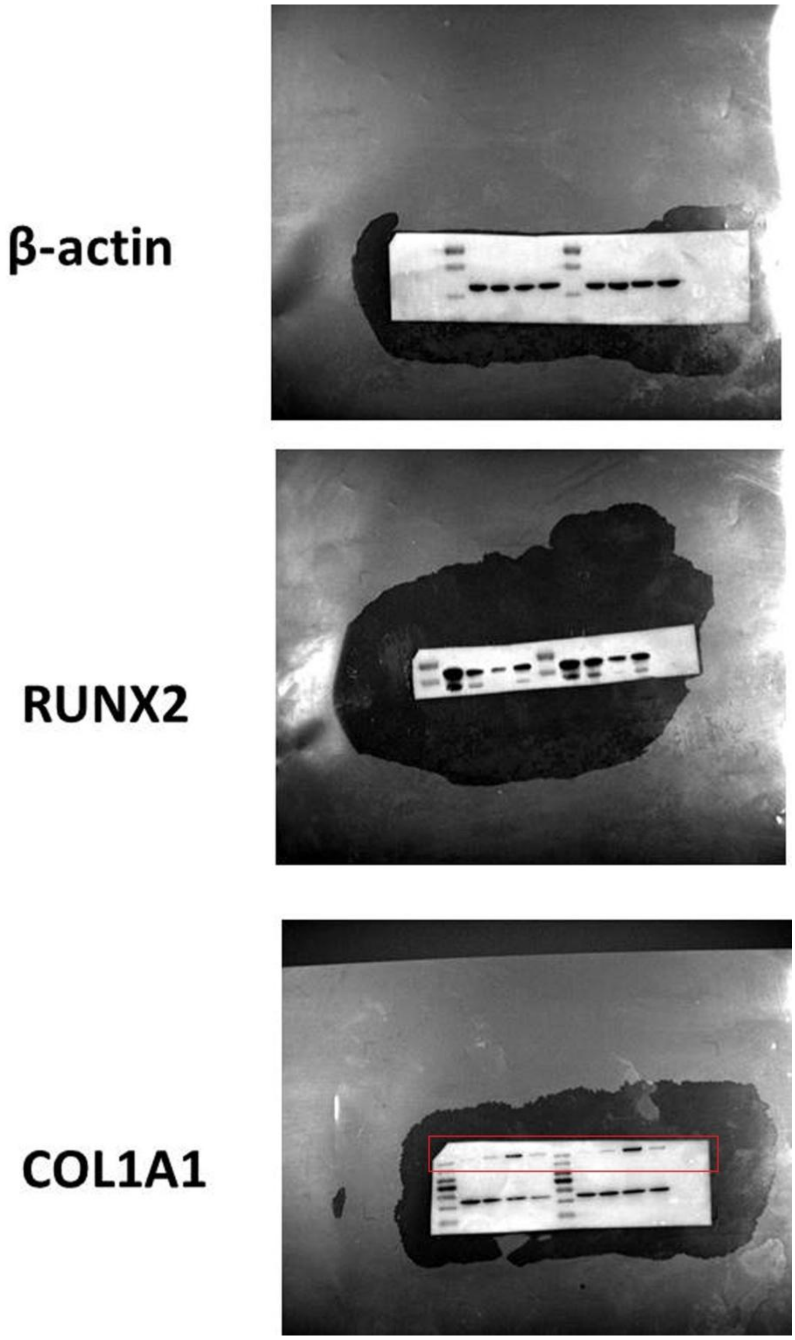

Supplementary figure 1

The RUNX2 and COL1A1 levels detected by western blot. The red rectangle in the bottom image represented COL1A1expressed level.

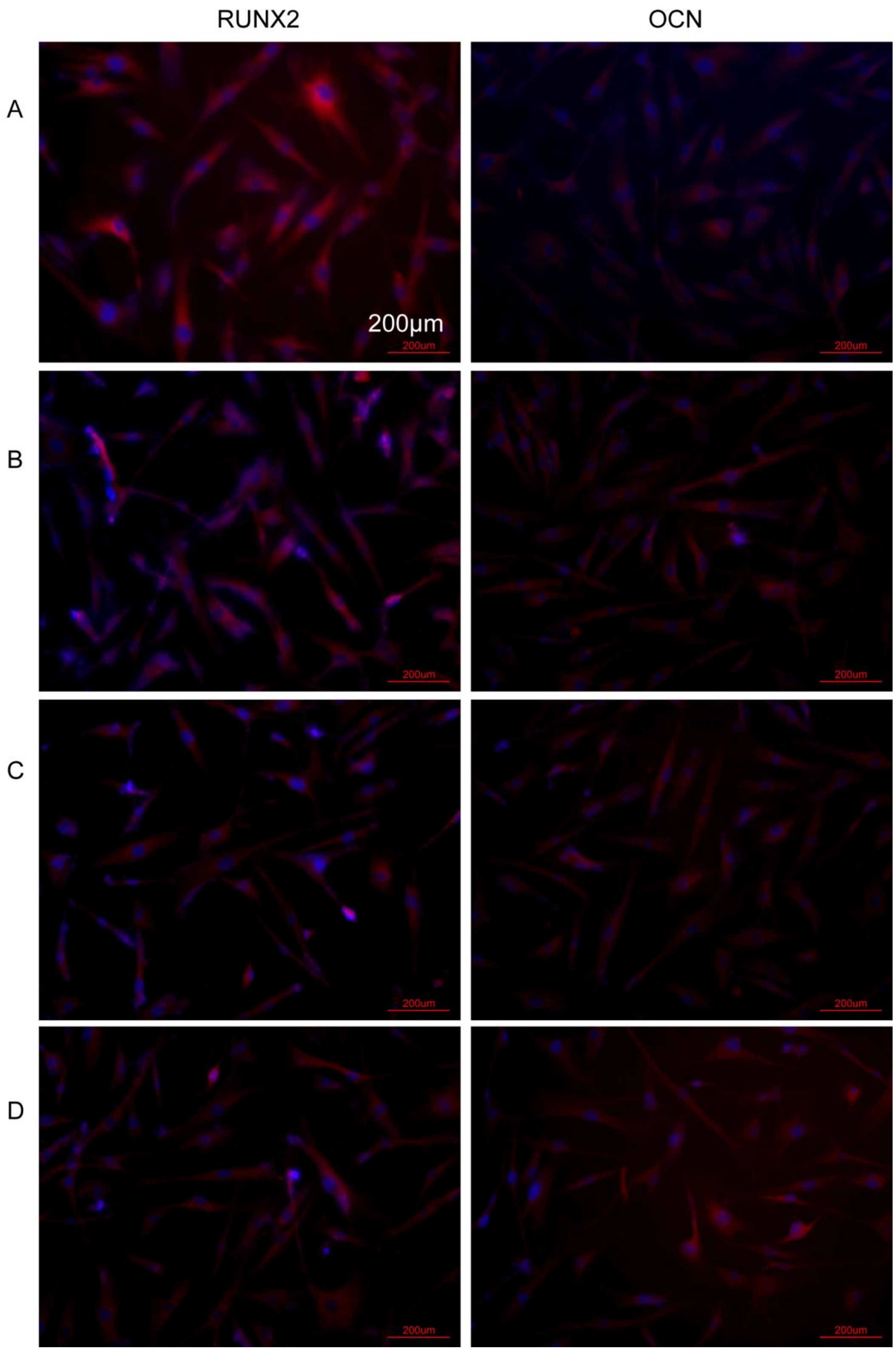

Supplementary figure 2

The immunohistochemical results of PGS-g-M scaffolds (A), and PGS-M-n-HA-0.4 (B), PGS-M-n-HA-0.5 (C), and PGS-M-n-HA-0.6 composite scaffolds (D).

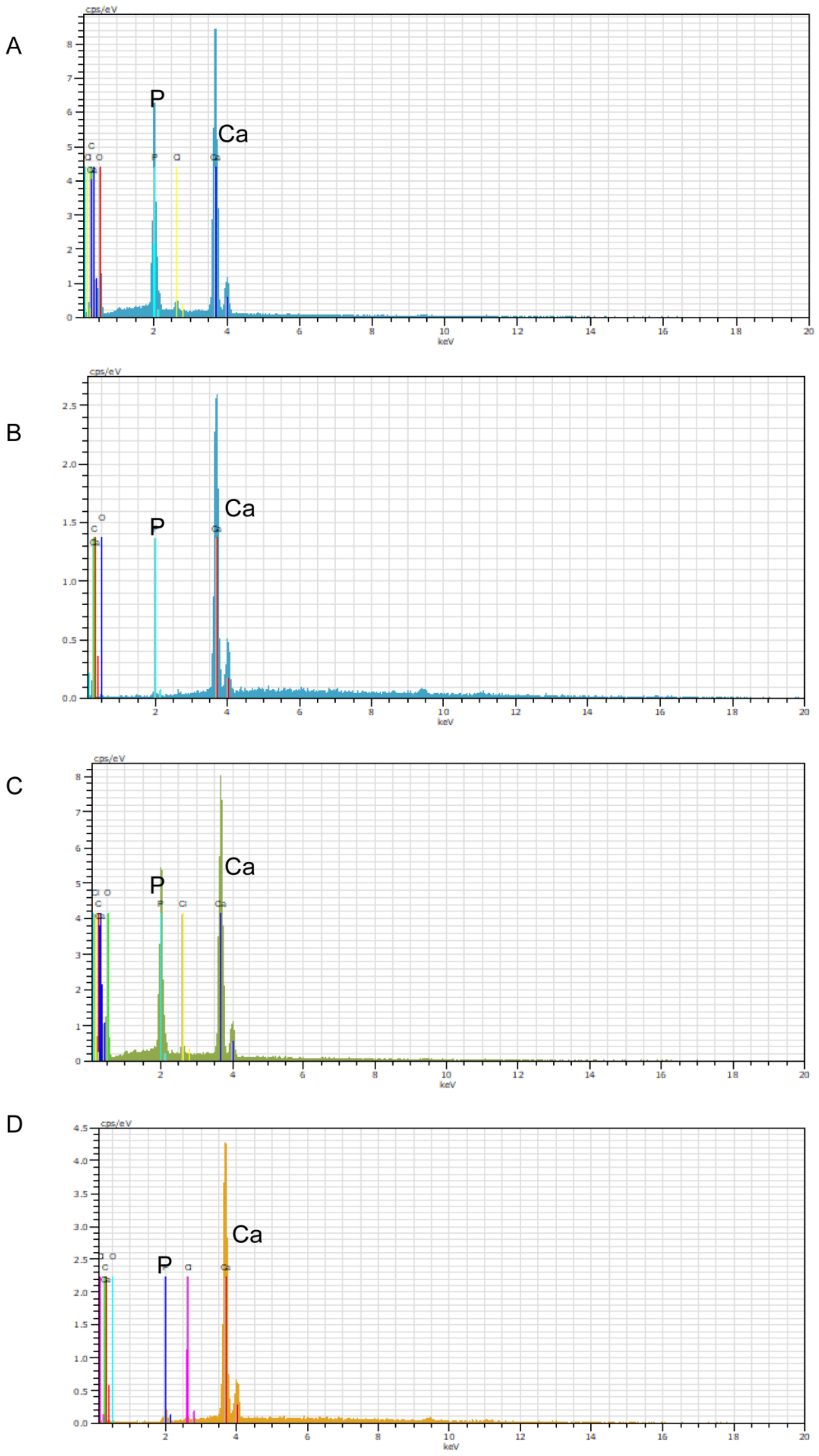

A
B
C
D
cps/eV
keV
P
Ca
C
O
Cl

Supplementary figure 3

The Ca-P chemical element analysis on the surface of the four scaffolds by energy-dispersive X-ray spectroscopy (EDS) at 4 weeks. PGS-g-M scaffolds (A), and PGS-M-n-HA-0.4 (B), PGS-M-n-HA-0.5 (C), and PGS-M-n-HA-0.6 composite scaffolds (D).
